# Acute Kidney Injury in Critically Ill Vascular Surgery Patients is Common and Associated with Increased Mortality

**DOI:** 10.3389/fsurg.2015.00008

**Published:** 2015-03-09

**Authors:** Donald G. Harris, Grace Koo, Michelle P. McCrone, Adam S. Weltz, William C. Chiu, Rajabrata Sarkar, Thomas M. Scalea, Jose J. Diaz, Matthew E. Lissauer, Robert S. Crawford

**Affiliations:** ^1^Department of Surgery, University of Maryland School of Medicine, Baltimore, MD, USA; ^2^R Adams Cowley Shock Trauma Center, University of Maryland School of Medicine, Baltimore, MD, USA; ^3^Department of Surgery, Rutgers – Robert Wood Johnson Medical School, New Brunswick, NJ, USA

**Keywords:** acute kidney injury, renal failure, vascular surgical procedures, perioperative outcomes, surgical critical care

## Abstract

**Introduction:** Vascular surgery patients have multiple risk factors for renal dysfunction, but acute kidney injury (AKI) is poorly studied in this group. The purpose of this study was to define the incidence, risk factors, and outcomes of AKI in high-risk vascular patients.

**Methods:** Critically ill vascular surgery patients admitted during January–December 2012 were retrospectively analyzed with 1-year follow-up. The endpoint was AKI by established RIFLE creatinine criteria. The primary analysis was between patients with or without AKI, with secondary analysis of post-operative AKI. Outcomes were inpatient and 1-year mortality, inpatient lengths of stay, and discharge renal function.

**Results:** One-hundred and thirty six vascular surgery patients were included, representing 27% of all vascular surgery admissions during the study period. Sixty-five (48%) developed AKI. Independent global risk factors for AKI were diabetes, increasing critical illness severity, and sepsis. While intraoperative blood loss and hypotension were associated with subsequent renal dysfunction, post-operative AKI rates were similar for patients undergoing aortic, carotid, endovascular, or peripheral vascular procedures. All RIFLE grades of AKI were associated with worse outcomes. Overall, patients with AKI had significantly increased short- and long-term mortality, longer inpatient lengths of stay, and worse discharge renal function.

**Conclusion:** AKI is common among critically ill vascular surgery patients. Importantly, the type of surgical procedure appears to be less important than intra- and perioperative management in determining renal dysfunction. Regardless of its severity, AKI is a clinically significant complication that is associated with substantially worse patient outcomes.

## Introduction

Acute kidney injury (AKI, characterized by a serum creatinine 50% above baseline or oliguria) is a common perioperative complication in surgical patients (1–4), and is among the most frequent forms of organ dysfunction among the critically ill (5–7). For surgical patients, AKI is associated with substantially worse outcomes, including longer inpatient lengths of stay and increased short- and long-term mortality (1–4, 8). Although morbidity and mortality is highest for patients with severe renal dysfunction (3, 9–11), even transient and relatively minor increases in serum creatinine are associated with worse outcomes (1, 11–15).

Vascular surgery patients have unique risk factors for AKI. High-risk comorbidities such as diabetes, hypertension, and tobacco use are common among vascular patients (15–17), and aortic cross-clamping, contrast-induced nephropathy, and cholesterol embolization are frequent renal hazards within this group (12, 18, 19). As a result, nearly all patients undergoing aortic surgery have at least biochemical renal dysfunction as demonstrated by albuminuria (20), and clinically significant AKI occurs in up to 25% (20, 21). While AKI is well studied in select subgroups, such as after aortic surgery (12, 20, 21), few reports have evaluated AKI in a broad group of vascular patients (22). As such, the epidemiology, risk factors, and outcomes that may be important and specific to these patients remain undefined.

Critically ill surgical patients have the highest risk for renal dysfunction (14, 23). As such, patients requiring surgical intensive care unit (SICU) admission represent an ideal, high-risk population to study AKI. To investigate the incidence, risk factors, and outcomes of AKI among critically ill vascular surgery patients, we performed a subgroup analysis of AKI in vascular surgery patients from a 1-year SICU cohort (1). We hypothesized that AKI is common within this population, associated with specific global and intraoperative risk factors, and is associated with worse patient outcomes.

## Materials and Methods

This study of AKI in critically ill vascular surgery patients was a retrospective analysis, and was approved by the University of Maryland, Baltimore Institutional Review Board. As a subgroup analysis of a previously described SICU cohort (1), the purpose of this report was to serve as a distinctly separate focus on AKI among critically ill vascular surgery patients. Briefly, the original cohort of SICU admissions during January–December 2012 was identified from a prospectively maintained Acute Physiology and Chronic Health Evaluation (APACHE) IV database (Cerner, Kansas City) (24, 25). Clinical data was retrospectively obtained from the database and by chart review, and 1-year post-discharge vital status was determined from the Social Security Death Index (1). For this study, additional operative, transfusion, and pharmacologic data specific to the vascular surgery patients were abstracted from clinical records.

Vascular surgery patients are admitted to the SICU on an individual basis based on the need for critical care support, frequent monitoring, administration of critical medications, or other intensive care unit level care; there are no protocols or pathways that include routine SICU admission. Institutional practices include routine hydration with balanced crystalloid infusions for patients who receive intravenous contrast and are assessed as being at risk for contrast nephropathy. Intravenous bicarbonate infusions or *N*-acetylcysteine are used as adjuncts at the discretion of the treating team. In the setting of myoglobinemia, aggressive hydration, diuresis, alkalinization, and potential renal support is performed by the critical care service. Renal replacement therapy is provided either continuously by the critical care service or intermittently by consulting nephrologists.

Baseline renal function was defined as the lowest serum creatinine (milligrams per deciliter) of: within 1 year prior to admission; at hospital or SICU admission; or, for patients without chronic kidney dysfunction, the Modification of Diet in Renal Disease equation solved for creatinine based on a glomerular filtration rate of 75 mL/min/1.73 m^2^ (1, 9, 26). Patients with a single inpatient creatinine value, on chronic dialysis or who had prior AKI, nephrectomy, or organ transplant were excluded. The primary endpoint was inpatient AKI, as diagnosed and classified according to RIFLE creatinine criteria (increase from baseline: risk ≥1.5×, injury ≥2×, and failure ≥3×) (26). Renal recovery was defined as a sustained decreasing creatinine below a patient’s AKI – risk threshold for more than 24 h without renal replacement therapy (1, 26). Medication use, comorbidities, and the presence of sepsis were defined by clinical documentation and APACHE IV data. Emergency status was defined as a non-elective hospital admission or need for emergency surgical intervention.

The primary analysis was between vascular surgery patients with or without inpatient AKI. To evaluate surgical factors associated with AKI, a secondary analysis comparing vascular patients with or without post-operative AKI was performed. Outcomes included inpatient and 1-year mortality, SICU and hospital lengths of stay, and discharge creatinine. Two-sided Fisher exact or Pearson chi-square tests were used to compare categorical data. Unpaired Student’s *t*-test was used to analyze normally distributed continuous data, which was reported as mean ± standard deviation. Mann–Whitney *U* test was used to compare non-normally distributed data, which was reported as median and interquartile range. Factors associated with AKI on univariate analysis with a *P* value <0.10 were further assessed by multivariate logistic regression with correction for age and gender, with results given as odds ratio (OR) and 95% confidence intervals. A *P* value <0.05 was accepted as statistically significant for all analyses.

## Results

There were 136 vascular surgery patients within the SICU cohort, representing 22% of the SICU cohort and 27% of all vascular surgery admissions during the period. Eighty-three (61%) of the patients were male, mean age was 65 ± 15 years, and APACHE III score was 50 ± 20 (Table 1), representing significant critical illness severity. Compared to vascular patients not requiring SICU admission, the study group had more acute limb ischemia (14 vs. 7%, *P* = 0.02), were more likely to receive operative management (91 vs. 74%, *P* < 0.0001), and underwent more aortic procedures (35 vs. 7%, *P* < 0.0001).

**Table 1:** **Characteristics of vascular surgery SICU patients**.

	All patients, *n* = 136	No AKI, *n* = 71	AKI, *n* = 65	*P*
**Demographics**
Age, years ± SD	65 ± 15	63 ± 16	67 ± 12	0.07
Male, *n* (%)	83 (61)	47 (66)	36 (55)	0.20
Black, *n* (%)	53 (39)	28 (39)	25 (38)	0.91
Baseline creatinine, mg/dL ± SD	0.93 ± 0.50	0.90 ± 0.44	0.96 ± 0.56	0.83
Chronic kidney disease, *n* (%)	18 (13)	10 (14)	8 (12)	0.76
COPD, *n* (%)	26 (19)	13 (18)	13 (20)	0.80
Diabetes mellitus, *n* (%)	40 (29)	16 (23)	24 (37)	0.07
Hypertension, *n* (%)	86 (63)	43 (61)	43 (66)	0.50
Peripheral arterial disease, *n* (%)	35 (26)	19 (27)	11 (17)	0.17
**Inpatient factors**
Emergency status, *n* (%)	73 (54)	38 (54)	35 (54)	0.97
APACHE III score ± SD	50 ± 20	44 ± 18	56 ± 20	0.0002
Surgical management, *n* (%)	121 (89)	63 (89)	58 (89)	0.93
Critical care and perioperative management
Mechanical ventilation	84 (62)	34 (48)	50 (77)	0.001
Days, median (IQR)	1 (0–3)	0 (0–2)	2 (1–6)	<0.0001
Transfusion, *n* (%)	89 (65)	38 (54)	52 (80)	0.001
Sepsis, *n* (%)	13 (10)	1 (1)	12 (18)	0.001
ACEI/ARB use, *n* (%)	40 (29)	28 (39)	12 (18)	0.007
Myoglobinemia >500 ng/mL, *n* (%)	18 (13)	12 (17)	6 (9)	0.21

*P value denotes comparison to AKI and non-AKI subgroups*.

*AKI, acute kidney injury; COPD, chronic obstructive pulmonary disease; APACHE, acute physiology and chronic health evaluation; ACEI, angiotensin-converting enzyme inhibitor; ARB, angiotensin receptor blocker*.

Acute kidney injury was common, and occurred in 65 (48%) critically ill vascular patients (Table 2), a rate similar to other patients in the SICU cohort (231/488, 47%; *P* = 0.92). Most events (39, 60%) were within the RIFLE – risk category, representing relatively mild renal dysfunction. Of the patients who recovered, approximately one-third developed recurrent kidney injury during their index hospitalization.

**Table 2:** **Acute kidney injury event characteristics**.

	AKI, *n* = 65
Present on admission, *n* (%)	22 (34)
Post-operative, *n* (%)	30 (46)
Interval, median days (IQR)	1 (1–6)
Peak creatinine, mg/dL ± SD	2.03 ± 1.22
RIFLE – risk, *n* (%)	39 (60)
RIFLE – injury, *n* (%)	14 (22)
RIFLE – failure, *n* (%)	12 (18)
Duration, median days (IQR)	3 (2–5)
Renal replacement therapy, *n* (%)	8 (12)
Renal recovery, *n* (%)	47 (72)
Recurrent kidney injury, *n* (%)	17 (36)

*AKI, acute kidney injury; RIFLE, risk, injury, failure, loss, end-stage renal failure acute kidney injury classification*.

Higher critical illness severity, mechanical ventilation, sepsis, and blood product transfusion were associated with AKI on univariate analysis (Table 1). In particular, rates of AKI increased with the total number of transfusions. While 28% of patients who received no transfusions developed AKI, among transfusion quartiles (1–3, 4–7, 8–10, and 11–41 total blood products), rates of AKI were 43, 46, 68, and 74% (*P* = 0.001). With multivariate analysis, diabetes (OR 1.3, 1.1–1.5), APACHE III score >50 (OR 1.3, 1.1–1.6), and sepsis (OR 1.4, 1.1–1.9) remained independently associated with developing AKI (Table S1 in Supplementary Material). Other characteristics, including age, baseline renal function, non-diabetes comorbidities, need for surgery, and significant myoglobinemia were not associated with renal dysfunction.

Among patients admitted with normal renal function, inpatient administration of an angiotensin-converting enzyme inhibitor or angiotensin II receptor blocker (which was determined by the critical care or vascular surgery team on a per-patient basis) was associated with a lower risk for developing AKI (30 vs. 54%, *P* = 0.01) on univariate analysis. In particular, while pre-admission treatment was itself not associated with AKI (43 vs. 35%, *P* = 0.35), among patients treated prior to admission, continued inpatient therapy was associated with a lower rate of renal dysfunction than if the medication was discontinued (33 vs. 69%, *P* = 0.01). No other potentially modifiable variable was associated with lower rates of AKI.

Rates of AKI were similar regardless of surgical vs. non-operative management (Table 1). Similarly, AKI rates were similar among patients who underwent aortic, carotid, peripheral vascular, endovascular, and other procedures (45–52%; not shown). Thirty patients developed renal dysfunction post-operatively at a median of 1 (1–6 days) after surgery (Table 2); these patients represented 46% of patients with AKI and 32% of patients who underwent surgery with normal renal function (Table 3). The category of procedure did not contribute to post-operative AKI, nor did aortic cross-clamp duration for open aortic surgery. Markers of intraoperative blood loss and hypotension were associated with subsequent renal dysfunction. After multivariate analysis of potential intraoperative factors, the need for two or more vasopressors (OR 2.2, 1.1–4.2) and blood loss >1000 mL (OR 1.4, 1.1–1.8) were each associated with post-operative AKI; male gender was associated with less risk for post-operative AKI (OR 0.8, 0.7–1.0; Table S1 in Supplementary Material).

**Table 3:** **Procedural and intraoperative factors of patients with or without post-operative AKI**.

	No AKI, *n* = 64	Post-op. AKI, *n* = 30	*P*
Elective case, *n* (%)	48 (75)	22 (73)	0.86
ASA classification	3 (3–4)	3 (3–4)	0.32
Procedural
Open aortic, *n* (%)	17 (27)	9 (30)	0.73
Cross-clamp time, min (IQR)	62 (47–103)	79 (67–92)	0.89
Peripheral vascular, *n* (%)	21 (33)	12 (40)	0.50
Endovascular, *n* (%)	8 (12)	4 (13)	1.00
Carotid, *n* (%)	5 (8)	2 (7)	1.00
Contrast exposure, *n* (%)	24 (38)	12 (40)	0.82
Intraoperative
Estimated blood loss, mL ± SD	700 ± 1000	1400 ± 2400	0.005
Transfusion, *n* (%)	31 (48)	22 (73)	0.02
Total blood products, median (IQR)	0 (0–3)	2 (0–4)	0.03
Lowest base excess, mEq/L	−1.9 ± 3.3	−3.3 ± 4.5	0.14
Vasopressor use, *n* (%)	3 (5)	4 (13)	0.14
≥2 Agents, *n* (%)	0	3 (10)	0.01
Hourly urine output, mL ± SD	220 ± 125	200 ± 140	0.64

*AKI, acute kidney injury; ASA, American Society of Anesthesiologists physical status classification*.

**Figure 1:**
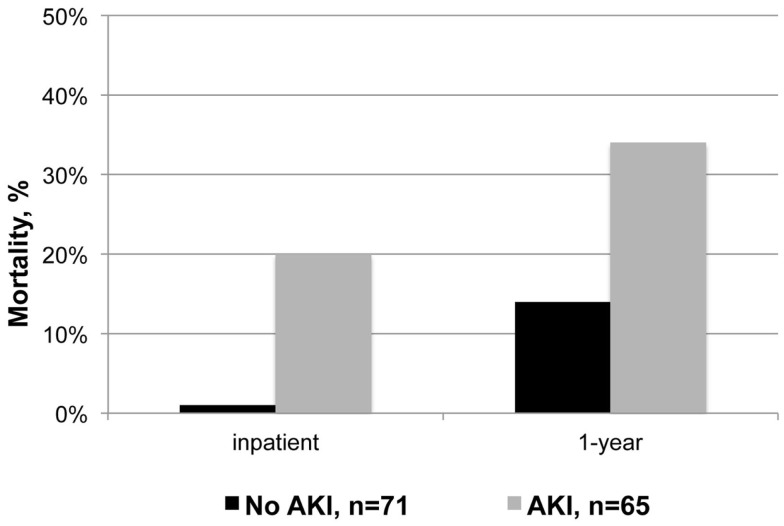
**Inpatient and 1-year mortality after acute kidney injury**.

Patients who developed AKI had significantly higher mortality during their index hospitalization and within 1-year of discharge (relative risk 14 and 2, respectively; Figure 1 and Table 4). Despite experiencing relatively mild renal dysfunction, even patients with RIFLE – risk AKI had significantly higher inpatient (18 vs. 1%, *P* = 0.003) and 1-year (31 vs. 14%, *P* = 0.04) than patients without AKI. Mortality rates appeared to increase with the severity of renal dysfunction (inpatient: risk 18% vs. failure 42%, *P* = 0.09; 1-year: inpatient 31 vs. 67%, *P* = 0.04). Finally, AKI patients had significantly longer SICU and hospital lengths of stay, and worse discharge renal function. As noted in Table 2, 28% of patients with AKI did not recover to a normal renal function.

**Table 4:** **Patient outcomes**.

	No AKI, *n* = 71	AKI, *n* = 65	*P*
Inpatient mortality, *n* (%)	1 (1)	13 (20)	<0.0001
1-year mortality, *n* (%)	10 (14)	22 (34)	0.007
SICU LOS, days (IQR)	3 (2–5)	5 (3–8)	0.0006
Hospital LOS, days (IQR)	9 (5–14)	16 (10–28)	<0.0001
Discharge creatinine, mg/dL ± SD	0.92 ± 0.49	1.47 ± 1.15	0.0002

*AKI, acute kidney injury; SICU, surgical intensive care unit; LOS, length of stay*.

## Discussion

This is one of the first studies to investigate AKI in a broad, high-risk group of vascular surgery patients. Renal dysfunction is common among vascular patients who require SICU admission, regardless of their primary surgical management, and is associated with significantly higher short- and long-term mortality, greater resource use, and worse discharge renal function. While outcomes are worse with higher AKI grades, even relatively minor renal dysfunction contributes to worse outcomes, which highlights the importance of optimizing prevention and management of AKI. As such, this study provides important insight into renal dysfunction among vascular surgery patients.

Consistent with prior studies of surgical and critically ill patients, diabetes, illness severity, and sepsis are global risk factors for AKI in vascular surgery patients (2–5, 9, 10, 15, 23, 27). Other comorbidities, such as hypertension, peripheral arterial disease, and major surgery have been associated with greater risk for renal dysfunction (3, 8, 15, 27). While these may contribute to AKI in vascular surgery *patients*, within this *population*, they may be relatively less important given their high prevalence (17). Both intraoperative and overall blood product transfusions were associated with AKI. While these were not independent factors after multivariate analysis adjusting for potential confounders and may reflect disease severity, there was a clear dose–response relationship between transfusion and AKI. Other studies have implicated transfusion as an independent risk factor for AKI, distant organ dysfunction, and worse patient outcomes (12, 21, 28–32). While the mechanism is unclear, blood product storage and administration may result in deleterious inflammatory and immunologic changes that contribute to organ dysfunction (31, 32).

Interestingly, in this cohort, the type of index procedure was not associated with AKI. Historically, open aortic surgery, particularly supra-renal procedures, has been associated with significant rates of renal dysfunction (20, 21, 33), particularly relative to other vascular interventions (22). Additionally, patients presenting with acute limb ischemia are at higher risk for AKI (22), likely due to the effects of rhabdomyolysis and ischemia-reperfusion injury (34). However, our data suggest that within this population of critically ill patients, comorbidities, intraoperative events, and post-operative factors define patterns of AKI. Although not defined in detail, the factors contributing to SICU admission and critical illness severity also likely contribute. Potentially, risk may be defined by perioperative stratification to optimize patient selection and management, and identify opportunities to modify selected risk factors.

By preventing the physiologic renal arteriolar constriction needed to maintain glomerular perfusion pressure, angiotensin-converting enzyme inhibitor and angiotensin II receptor blockers may contribute to acute renal dysfunction. However, inpatient treatment with an angiotensin II pathway antagonist was associated with a protective effect in this study. While there are mixed results of these agents on post-operative AKI in patients with cardiovascular risk factors (35, 36), they have an established protective effect in patients at risk for chronic renal disease (37, 38) and may have a role in preventing contrast nephropathy (39). Our data suggest a beneficial effect among vascular surgery patients, particularly those already taking an angiotensin II pathway antagonist. As such, further investigation of the role of these agents in modulating or protecting against acute renal function is required.

Open aortic surgery is traditionally associated with increased risk for post-operative renal dysfunction, and these patients are frequently exposed to intraoperative blood loss, hypotension, and transfusions (20–22, 33). However, in this group of critically ill vascular surgery patients, global and post-operative AKI rates were similar regardless of the classification of the type of procedure. This may represent selection bias since our institutional practice includes routine SICU admission after aortic surgery, but even patients undergoing lower-risk procedures clearly have high rates of AKI if they become critically ill. As such, while specific operative factors affect AKI rates among patients undergoing aortic surgery (20–22, 33), perioperative management may be just as critical for vascular surgery patients as a population.

Acute kidney injury in critically ill vascular surgery patients was associated with significantly worse mortality and other outcomes, as demonstrated in similar studies (9, 12, 20, 30). While AKI may be a marker of worse illness severity, it can contribute directly to multi-organ dysfunction and mortality by enhancing systemic inflammation and contributing to distant organ injury (7, 40). Supporting the clinical significance of relatively minor creatinine changes (13–15), even patients with RIFLE – risk AKI had significantly increased mortality. AKI of any severity should be recognized and managed as a high-risk complication. Among vascular surgery patients, prevention and treatment may include ensuring optimal resuscitation, limiting contrast exposure, aggressively treating sepsis, and avoiding unnecessary blood product transfusions.

The retrospective design of this study limited the ability to fully account for potential confounders of important risk factors such as patient selection, inpatient medical therapies, transfusions, and procedural details. The use of RIFLE creatinine thresholds alone to define and stage AKI may have missed events meeting urine output criteria for renal dysfunction. Similarly, although critically ill patients have the greatest risk for AKI, by only analyzing vascular patients requiring SICU admission, we may have missed a major disease burden among those not admitted to the SICU. Certain practices which may mitigate AKI, such as intravenous hydration to prevent contrast nephropathy, were not assessed in this study. While the sample represented a range of vascular pathology and procedures, features potentially unique to our patients and practice may limit the generalizability of these findings. Despite these limitations, incidence and impact of AKI among vascular surgery patients is clear, and should warrant a prospective, multicenter study to better define modifiable opportunities for prevention and treatment.

## Conclusion

Acute kidney injury is common and clinically significant complication in vascular surgery patients who require SICU admission, regardless of the primary surgical management. Patients who develop renal dysfunction have significantly greater mortality, even with relatively minor AKI. While traditional risk factors for AKI are common among these patients, diabetes is independently associated with higher rates of renal dysfunction. Intraoperative blood loss and hypotension, transfusions, critical illness severity, and sepsis are important and potentially modifiable risk factors.

## Conflict of Interest Statement

While the authors have no conflicts of interest relevant to this manuscript, Robert S. Crawford does educational consulting for Cook Medical and Medtronic.

## Supplementary Material

The Supplementary Material for this article can be found online at http://www.frontiersin.org/Journal/10.3389/fsurg.2015.00008/abstract

## References

[1] Harris DG McCrone MP Koo G Weltz AS Chiu WC Scalea TM Epidemiology and outcomes of acute kidney injury in critically ill surgical patients. J Crit Care (2015) 30(1):102–6. 10.1016/j.jcrc.2014.07.028 25171816

[2] Bihorac A Brennan M Ozrazgat-Baslanti T Bozorgmehri S Efron PA Moore FA National surgical quality improvement program underestimates the risk associated with mild and moderate postoperative acute kidney injury. Crit Care Med (2013) 41:2570–83. 10.1097/CCM.0b013e31829860fc 23928835 PMC3812338

[3] Bihorac A Yavas S Subbiah S Hobson CE Schold JD Gabrielli A Long-term risk of mortality and acute kidney injury during hospitalization after major surgery. Ann Surg (2009) 249:851–8. 10.1097/SLA.0b013e3181a40a0b 19387314

[4] Hobson CE Yavas S Segal MS Schold JD Tribble CG Layon AJ Acute kidney injury is associated with increased long-term mortality after cardiothoracic surgery. Circulation (2009) 119:2444–53. 10.1161/CIRCULATIONAHA.108.800011 19398670

[5] Gaieski DF Edwards JM Kallan MJ Carr BG. Benchmarking the incidence and mortality of severe sepsis in the United States. Crit Care Med (2013) 41:1167–74. 10.1097/CCM.0b013e31827c09f8 23442987

[6] Vincent JL de Mendonça A Cantraine F Moreno R Takala J Suter PM Use of the SOFA score to assess the incidence of organ dysfunction/failure in intensive care units: results of a multicenter, prospective study. Crit Care Med (1998) 26:1793–800. 10.1097/00003246-199811000-00016 9824069

[7] Wohlauer MV Sauaia A Moore EE Burlew CC Banerjee A Johnson J. Acute kidney injury and posttrauma multiple organ failure: the canary in the coal mine. J Trauma Acute Care Surg (2012) 72:373–80. 10.1097/TA.0b013e318244869b 22327979

[8] Kheterpal S Tremper KK Englesbe MJ O’Reilly M Shanks AM Fetterman DM Predictors of postoperative acute renal failure after noncardiac surgery in patients with previously normal renal function. Anesthesiology (2007) 107:892–902. 10.1097/01.anes.0000290588.29668.38 18043057

[9] Hoste EA Clermont G Kersten A Venkataraman R Angus DC De Bacquer D RIFLE criteria for acute kidney injury are associated with hospital mortality in critically ill patients: a cohort analysis. Crit Care (2006) 10:R73. 10.1186/cc4915 16696865 PMC1550961

[10] Cruz DN Bolgan I Perazella MA Bonello M de Cal M Corradi V North East Italian prospective hospital renal outcome survey on acute kidney injury (NEiPHROS-AKI): targeting the problem with the RIFLE criteria. Clin J Am Soc Nephrol (2007) 2:418–25. 10.2215/CJN.03361006 17699446

[11] Chertow GM Burdick E Honour M Bonventre JV Bates DW. Acute kidney injury, mortality, length of stay, and costs in hospitalized patients. J Am Soc Nephrol (2005) 16:3365–70. 10.1681/ASN.2004090740 16177006

[12] Ellenberger C Schweizer A Diaper J Kalangos A Murith N Katchatourian G Incidence, risk factors and prognosis of changes in serum creatinine early after aortic abdominal surgery. Intensive Care Med (2006) 32:1808–16. 10.1007/s00134-006-0308-1 16896848

[13] Lassnigg A Schmidlin D Mouhieddine M Bachmann LM Druml W Bauer P Minimal changes of serum creatinine predict prognosis in patients after cardiothoracic surgery: a prospective cohort study. J Am Soc Nephrol (2004) 15:1597–605. 10.1097/01.ASN.0000130340.93930.DD 15153571

[14] Uchino S Bellomo R Bagshaw SM Goldsmith D. Transient azotaemia is associated with a high risk of death in hospitalized patients. Nephrol Dial Transplant (2010) 25:1833–9. 10.1093/ndt/gfp624 20054022

[15] Goldberg A Kogan E Hammerman H Markiewicz W Aronson D. The impact of transient and persistent acute kidney injury on long-term outcomes after acute myocardial infarction. Kidney Int (2009) 76:900–6. 10.1038/ki.2009.295 19657321

[16] de Mendonça A Vincent JL Suter PM Moreno R Dearden NM Antonelli M Acute renal failure in the ICU: risk factors and outcome evaluated by the SOFA score. Intensive Care Med (2000) 26:915–21. 10.1007/s001340051281 10990106

[17] Nowygrod R Egorova N Greco G Anderson P Gelijns A Moskowitz A Trends, complications, and mortality in peripheral vascular surgery. J Vasc Surg (2006) 43:205–16. 10.1016/j.jvs.2005.11.002 16476588

[18] Scolari F Tardanico R Zani R Pola A Viola BF Movilli E Cholesterol crystal embolism: a recognizable cause of renal disease. Am J Kidney Dis (2000) 36:1089–109. 10.1053/ajkd.2000.19809 11096032

[19] Alamartine E Phayphet M Thibaudin D Barral F Veyret C Contrast medium-induced acute renal failure and cholesterol embolism after radiological procedures: incidence, risk factors, and compliance with recommendations. Eur J Intern Med (2003) 14:426–31 10.1016/j.ejim.2003.08.007 14614975

[20] Tallgren M Niemi T Pöyhiä R Raininko E Railo M Salmenperä M Acute renal injury and dysfunction following elective abdominal aortic surgery. Eur J Vasc Endovasc Surg (2007) 33:550–5. 10.1016/j.ejvs.2006.12.005 17276098

[21] Godet G Fleron M Vicaut E Zubicki A Bertrand M Riou B Risk factors for acute postoperative renal failure in thoracic or thoracoabdominal aortic surgery: a prospective study. Anesth Analg (1997) 85:1227–32. 10.1213/00000539-199712000-00009 9390585

[22] Black S Brooks M Naidoo M Wolfe J. Assessing the impact of renal impairment on outcome after arterial intervention: a prospective review of 1559 patients. Eur J Vasc Endovasc Surg (2006) 32:300–4. 10.1016/j.ejvs.2006.04.032 16781877

[23] Uchino S Kellum JA Bellomo R Doig GS Morimatsu H Morgera S Acute renal failure in critically ill patients. JAMA (2005) 294:813–8. 10.1001/jama.294.7.813 16106006

[24] Zimmerman JE Kramer AA McNair DS Malila FM. Acute physiology and chronic health evaluation (APACHE) IV: hospital mortality assessment for today’s critically ill patients. Crit Care Med (2006) 34:1297–310. 10.1097/01.CCM.0000215112.84523.F0 16540951

[25] Lissauer ME Galvagno SM Jr Rock P Narayan M Shah P Spencer H Increased ICU resource needs for an academic emergency general surgery service. Crit Care Med (2013) 42(4):910–7. 10.1097/CCM.0000000000000099 24335442

[26] Bellomo R Ronco C Kellum JA Mehta RL Palevsky P. Acute renal failure-definition, outcome measures, animal models, fluid therapy and information technology needs: the second international consensus conference of the acute dialysis quality initiative (ADQI) group. Crit Care (2004) 8:R204–12. 10.1186/cc2671 15312219 PMC522841

[27] Kheterpal S Tremper KK Heung M Rosenberg AL Englesbe M Shanks AM Development and validation of an acute kidney injury risk index for patients undergoing general surgery: results from a national data set. Anesthesiology (2009) 110:505–15. 10.1097/ALN.0b013e3181979440 19212261

[28] Karkouti K Wijeysundera DN Yau TM Callum JL Cheng DC Crowther M Acute kidney injury after cardiac surgery: focus on modifiable risk factors. Circulation (2009) 119:495–502. 10.1161/CIRCULATIONAHA.108.786913 19153273

[29] Miller PR Croce MA Kilgo PD Scott J Fabian TC. Acute respiratory distress syndrome in blunt trauma: identification of independent risk factors. Am Surg (2002) 68:845–50. 12412708

[30] Piffaretti G Mariscalco G Bonardelli S Sarcina A Gelpi G Bellosta R Predictors and outcomes of acute kidney injury after thoracic aortic endograft repair. J Vasc Surg (2012) 56:1527–34. 10.1016/j.jvs.2012.05.106 23058721

[31] Silverboard H Aisiku I Martin GS Adams M Rozycki G Moss M. The role of acute blood transfusion in the development of acute respiratory distress syndrome in patients with severe trauma. J Trauma Acute Care Surg (2005) 59:717–23. 10.1097/01.ta.0000174919.35240.21 16361918

[32] Corwin HL Gettinger A Pearl RG Fink MP Levy MM Abraham E The CRIT study: anemia and blood transfusion in the critically ill – current clinical practice in the United States. Crit Care Med (2004) 32:39–52. 10.1097/01.CCM.0000104112.34142.79 14707558

[33] Miller CC III Villa MA Achouh P Estrera AL Azizzadeh A Coogan SM Intraoperative skeletal muscle ischemia contributes to risk of renal dysfunction following thoracoabdominal aortic repair. Eur J Cardiothorac Surg (2008) 33:691–4. 10.1016/j.ejcts.2008.01.006 18261919

[34] Yassin MM Harkin DW Barros D’Sa AA Halliday MI Rowlands BJ. Lower limb ischemia-reperfusion injury triggers a systemic inflammatory response and multiple organ dysfunction. World J Surg (2002) 26:115–21. 10.1007/s00268-003-7093-6) 11898044

[35] Benedetto U Sciarretta S Roscitano A Fiorani B Refice S Angeloni E Preoperative angiotensin-converting enzyme inhibitors and acute kidney injury after coronary artery bypass grafting. Ann Thorac Surg (2008) 86:1160–5. 10.1016/j.athoracsur.2008.06.018 18805152

[36] Arora P Rajagopalam S Ranjan R Kolli H Singh M Venuto R Preoperative use of angiotensin-converting enzyme inhibitors/angiotensin receptor blockers is associated with increased risk for acute kidney injury after cardiovascular surgery. Clin J Am Soc Nephrol (2008) 3:1266–73. 10.2215/CJN.05271107 18667735 PMC2518804

[37] Lewis EJ Hunsicker LG Bain RP Rohde RD The effect of angiotensin-converting-enzyme inhibition on diabetic nephropathy. N Engl J Med (1993) 329:1456–62 10.1056/NEJM199311113292004 8413456

[38] Ravid M Savin H Jutrin I Bental T Katz B Lishner M. Long-term stabilizing effect of angiotensin-converting enzyme inhibition on plasma creatinine and on proteinuria in normotensive type II diabetic patients. Ann Intern Med (1993) 118:577–81. 10.7326/0003-4819-118-8-199304150-00001 8452322

[39] Gupta RK Kapoor A Tewari S Sinha N Sharma RK. Captopril for prevention of contrast-induced nephropathy in diabetic patients: a randomised study. Indian Heart J (1999) 51:521–6. 10721643

[40] White LE Chaudhary R Moore LJ Moore FA Hassoun HT. Surgical sepsis and organ crosstalk: the role of the kidney. J Surg Res (2011) 167:306–15. 10.1016/j.jss.2010.11.923 21324390 PMC3077465

