# Supplementary material for: Acute Kidney Injury in Critically Ill Vascular Surgery Patients is Common and Associated with Increased Mortality
Source: Front Surg. 2015 Mar 9;2:8. doi: 10.3389/fsurg.2015.00008 (PMC4353172; doi:10.3389/fsurg.2015.00008)
Supplement: Supplementary file 1 [file table_1.doc]

**Table S1.** Summary of multivariate analyses of risk factors for inpatient and postoperative AKI.

| **Risk Factors for Inpatient AKI** | | |  | **Risk Factors for Postoperative AKI** | | |
| --- | --- | --- | --- | --- | --- | --- |
| Inpatient Factor | Odds Ratio  (95% confidence interval) | *P* value |  | Intraoperative Factor | Odds Ratio  (95% confidence interval) | *P* value |
|  |  |  |  |  |  |  |
| Age > 70 years | 1.0 (0.9 – 1.2) | 0.87 |  | AGE > 70 | 1.1 (0.9 – 1.3) | 0.55 |
| Male | 0.9 (0.8 – 1.1) | 0.20 |  | Male | 0.8 (0.7 – 0.9) | 0.01 |
| APACHE III score> 50 | 1.3 (1.1 – 1.6) | 0.01 |  | Vasopressor | 1.0 (0.6 – 1.5) | 0.90 |
| Transfusion | 1.5 (0.8 – 2.9) | 0.18 |  | ≥ 2 vasopressors | 2.2 (1.1 – 4.2) | 0.02 |
| Mechanical ventilation | 1.2 (1.0 – 1.4) | 0.09 |  | Transfusions | 1.1 (0.8 – 1.3) | 0.70 |
| Sepsis | 1.4 (1.1 – 1.9) | 0.01 |  | Estimated blood loss > 1 liter | 1.4 (1.1 – 1.8) | 0.02 |
| Diabetes | 1.3 (1.1 – 1.5) | 0.01 |  |  |  |  |

Factors with associated with the specific outcome on univariate analysis with a *P* value < 0.10 were included for multivariable logistic regression analysis.
